# How a Paleogenomic Approach Can Provide Details on Bioarchaeological Reconstruction: A Case Study from the Globular Amphorae Culture

**DOI:** 10.3390/genes12060910

**Published:** 2021-06-11

**Authors:** Stefania Vai, Maria Angela Diroma, Costanza Cannariato, Alicja Budnik, Martina Lari, David Caramelli, Elena Pilli

**Affiliations:** 1 Department of Biology, University of Florence, 50122 Florence, Italy; mariaangela.diroma@unifi.it (M.A.D.); costanza.cannariato@unifi.it (C.C.); martina.lari@unifi.it (M.L.); david.caramelli@unifi.it (D.C.); elena.pilli@unifi.it (E.P.); 2 Department of Human Biology, Institute of Biological Sciences, Cardinal Stefan Wyszyński University, 01-938 Warsaw, Poland; alicja.budnik.uksw@gmail.com

**Keywords:** ancient DNA, palaeogenomics, Neolithic, multiple burial, kinship, phenotypic traits

## Abstract

Ancient human remains have the potential to explain a great deal about the prehistory of humankind. Due to recent technological and bioinformatics advances, their study, at the palaeogenomic level, can provide important information about population dynamics, culture changes, and the lifestyles of our ancestors. In this study, mitochondrial and nuclear genome data obtained from human bone remains associated with the Neolithic Globular Amphorae culture, which were recovered in the Megalithic barrow of Kierzkowo (Poland), were reanalysed to gain insight into the social organisation and use of the archaeological site and to provide information at the individual level. We were able to successfully estimate the minimum number of individuals, sex, kin relationships, and phenotypic traits of the buried individuals, despite the low level of preservation of the bone samples and the intricate taphonomic conditions. In addition, the evaluation of damage patterns allowed us to highlight the presence of “intruders”—that is, of more recent skeletal remains that did not belong to the original burial. Due to its characteristics, the study of the Kierzkowo barrow represented a challenge for the reconstruction of the biological profile of the human community who exploited it and an excellent example of the contribution that ancient genomic analysis can provide to archaeological reconstruction.

## 1. Introduction

Over the years, the study of ancient DNA study has proven valuable and essential for tracing migrations of historic and prehistoric individuals and groups. Advances in the sequencing and analysis of the genomes of both modern and ancient peoples have facilitated a number of breakthroughs in the understanding of human evolutionary history [1]. In this context, the archaeological excavations and retrieval of skeletal human remains represent a unique opportunity to study a population history and shed light on cultural changes and the lifestyles of our ancestors.

Archaeological excavations in the Żnin district (northwestern Poland), led by Professor Tadeusz Wiślański from the Institute of History and Material Culture of the Polish Academy of Sciences in Poznań, uncovered a Megalithic barrow tomb associated with the Globular Amphora culture (GAC); it contained the remains of several individuals. Through the integration of archaeological, anthropological, and molecular (mtDNA and nuDNA) data, and theories about the development of the GAC, our previous results [2,3] add nuance to the model of Late Neolithic gene flow from the Pontic steppes into Central Europe, showing that the eastern affinities of the GAC in the archaeological record reflect cultural influences rather than movements of people. During the study, archaeogenomic research permitted us to obtain information on past population dynamics, whereas biological traits (such as phenotypic traits) of each individual and relationships within the group in the burial site have not been investigated due to the fact that samples with presumed kinship relations were removed from our previous studies. However, beyond population genetics purposes, as recently proposed, for instance, by [4,5,6,7], obtaining more information on the individuals (such as sex estimation and phenotype descriptions of physical appearance and functional traits), and investigating the relationships among them could be important—in general and in particular for the Megalithic barrow of Kierzkowo—for better understanding the social structure and funerary rituals of ancient cultures and preserving and enhancing a specific archaeological site. In fact, the molecular characterisation of human remains can represent an important information source to describe past populations, also for educational purposes: genomic data can be integrated in archaeological and anthropological reconstruction in order to reach a wider public in contexts devoted to science and culture communication, such as museum exhibitions, archaeological parks, and documentaries.

Methods based solely on archaeological and anthropological assessments can sometimes be limited in establishing biological kinship and providing data on biological traits, above all, in contexts such as this Megalithic barrow tomb, in which, over time, anthropic action and post-depositional natural events had a strong impact on the ease of data analysis and interpretation.

Therefore, we decided, here, to reanalyse the molecular data of GAC from our previous studies, attempting to recover information, such as sex, phenotypic, and functional traits to provide individual characterisation, concentrating our attention, in particular, on closely related individuals (previously eliminated from the analysis) and their relations, with archaeological context, to obtain more information useful to reconstruct GAC funerary rituals and social organisation. The complexity of this archaeological site, characterised by a secondary multiple burial of fragmented and mixed human remains belonging to several individuals, represented a challenge for the reconstruction of the biological profile of this community and an excellent example of the contribution that aDNA analysis can provide to archaeological reconstruction. In this context, a molecular investigation turns out to be fundamental to integrate and confirm anthropological and archaeological data/hypotheses, and suggests further insight.

## 2. Materials and Methods

### 2.1. The Archaeological Site of Kierzkowo

**Figure 1:**
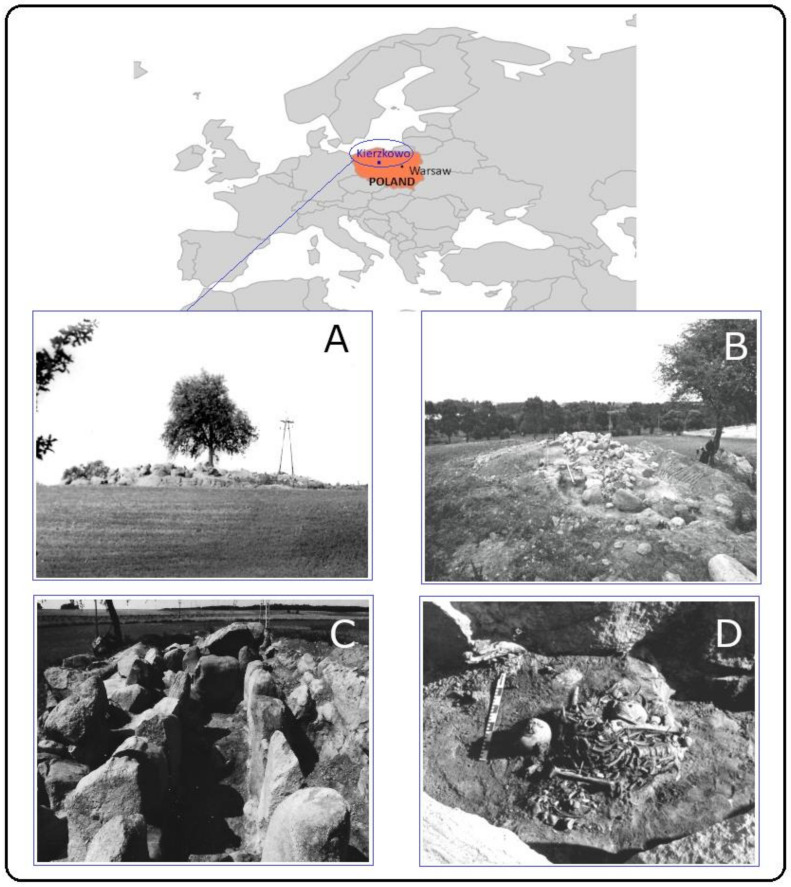
Geographic location and details of the site. Location of Kierzkowo site (Poland). (**A**) Megalithic barrow in Kierzkowo. (**B**) Megalithic barrow after removing the earth embankment. (**C**) Detail of the barrow: burial chamber. (**D**) Cluster of human Neolithic bones within the chamber. (**A**–**D**): photographed by Professor T. Wiślański; (**A**,**D**): fragments of photographs, adapted with permission from book [11]; (**B**,**C**): from the Archives of the Institute of Archaeology and Ethnology of the Polish Academy of Sciences.

The archaeological site of Kierzkowo is located in the Żnin district (Kuyavian-Pomeranian voivodeship, northwestern Poland, Figure 1) on top of a hill on the shore of Kierzkowski Lake. Pottery fragments belonging to characteristic decorated globular vessels [8,9,10,11], and a radiocarbon date on animal bone found in the site confirmed its attribution to the GAC period (4270 ± 40 BP calibrated to 2896 ± 19 cal BCE) [12].

The original barrow embankment was about 35 m long, 16–18 m wide, and 2 to 4 m high, overgrown by trees and bushes (Figure 1A). After removing the earth embankment, a stone structure approximately 22 m long, W–E, and 3 to 6 m wide, N–S, was revealed (Figure 1B). In the western part of this Megalithic tomb, there was a chamber about 10 m long, about 1.5 m wide, and of the same depth. The chamber was built of stone slabs with a height of about 1 m and a thickness of 0.5 to 0.8 m. Inside, a large boulder divided the chamber into two unequal parts. On the south side, a corridor lined with stones led to the chamber [11,13,14] (Figure 1C).

Within the chamber, Neolithic human bones gathered into two large clusters and a smaller one (at metres 3 and 4 and 5, Figure 1D). Some bones were also located under the large stone dividing the chamber, and much fewer bones were found outside of the chamber. In the two large bone gatherings, remains were stratified into seven layers, while in other locations, bones were in superficial layers. In many instances, human bones were mixed with animal bones with signs of dismemberment [11]. The presence of domesticated animal remains testify that animal husbandry was the dominant form of economy. Individuals buried in Kierzkowo, indeed, being people of the GAC, belonged to the set of agriculture-based economies with a dominance of pastoralism. They were nomadic people with unstable settlement patterns. The megalithic tombs may have been important central points or boundaries of the territory used by the travelling human group [8,15,16,17]. Consumption of milk and animal meat is attested by the analysis of lipid residues in ceramic vessels from the barrow. Carbon (δ^13^C) and nitrogen (δ^15^N) isotopes were used to reconstruct the diet of the individuals from Kierzkowo. The δ^15^N values in bone collagen were slightly higher in adult men than in adult women. This could indicate a slightly better access of men to valuable animal products, but this differentiation is very small and requires further research [11].

**Figure 2:**
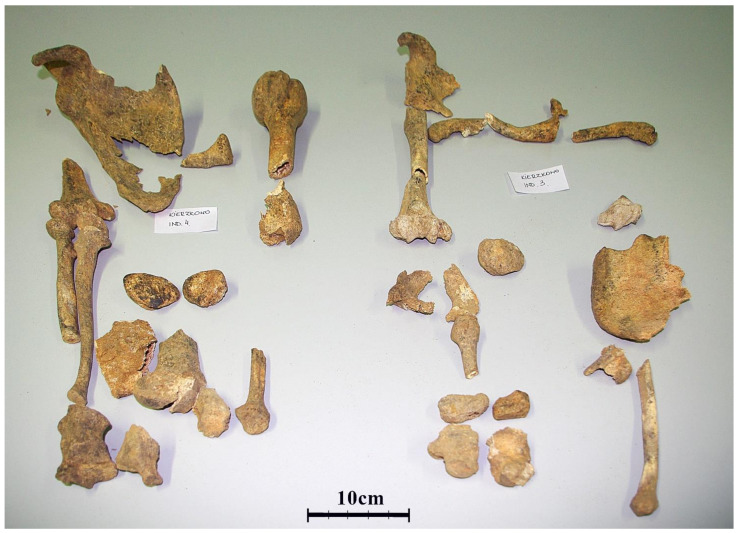
Fragments of bones of two individuals partly arranged in anatomical order. Bone fragments were tentatively attributed to individuals considering sex and age estimations, side, size, robustness, surface relief, colour, and state of preservation. Photo by Dr Andrzej Długoński.

Most of the human remains found in Kierzkowo were highly fragmented and mixed, without anatomical order, placed in the barrow as secondary interment (Figure 1D and Figure 2). Altogether, 428 fragments of various parts of the skeleton were recovered, 94.2% of them from the burial chamber. These fragments were studied for taphonomy and morphological traits. Type of bone, skeletal area, side of the body, size of a fragment, and its robustness surface markings were taken into account. Furthermore, the degree of bone deterioration, traces of plant roots and eventual activity of invertebrates, bone colour and its alterations, types of breaks and soil in which they were preserved were also considered. Wherever possible, sex and age at the death of individuals were estimated. Preliminary descriptions and anthropological analyses were made many years ago [18]. At present, all bone fragments were inspected again and revisions of diagnoses (such as estimations of sex and age) were performed using newer diagnostic methods.

Evaluating sex and age estimations, the origin from the left or right side of the skeleton, the number of paired bones in the skeleton, size, robustness, surface relief, and also colour and state of preservation, bone fragments were assembled into individual skeletons (Figure 2). It needs to be stressed that the application of only morphological criteria does not produce complete certainty about bone allocation to the same individual. The Ockham’s razor principle was used for allocating bone fragments to individuals in order to not multiply numbers of individuals beyond the necessary minimum. Finally, we estimated that in the barrow there were at least 27 persons buried [11]. Both sexes and ages ranging from newborns to elders were represented.

### 2.2. The Analysed Samples

Seventeen bone remains from Kierzkowo were chosen for genetic analysis as they were attributed to the same number of putative different individuals (Table 1).

The primary reports of the molecular data obtained from these samples were previously published by [2,3], where population genetics methods were applied to these and other samples to investigate population dynamics during the Neolithic period in Europe. Complete or almost complete mtDNA consensus sequences were available in GenBank with accession numbers MF114211-MF114224. Sequence alignments of nuclear DNA (nuDNA) reads in BAM (Binary Sequence Alignment/Map) format were retrieved for eight individuals from the European Nucleotide Archive (ENA) under accession numbers ERS2040835-ERS2040840, ERS2040844, and ERS2040845.

In this paper, both mtDNA and nuDNA data were object to a new kind of analysis with a specific focus on the single community buried in the Kierzkowo site.

### 2.3. Mitochondrial Haplotype and Haplogroup Analysis

The available mtDNA consensus sequences were reanalysed to estimate haplogroups and phylogenetic relationships between haplotypes.

Different from our previous study [3], HaploGrep 2.0 [19] was used for haplogroup assignment based on PhyloTree Build 17 [20]. Polymorphisms according to the revised Cambridge reference sequence (rCRS) were annotated and showed in a graphical phylogenetic tree obtained by HaploGrep 2.0.

Misincorporation rates at read termini and average fragment length were calculated by MapDamage2.0 [21] to describe the damage pattern of the mtDNA molecules. Modern human contamination at mitochondrial level was estimated by ContamMix [22]. Default parameters were used for these analyses.

### 2.4. Sex Identification

Sex identification of all samples for which nuDNA data were available was performed using the guidelines suggested by Skoglund et al. [23] (v0.4). A python script was applied to the read alignments in BAM format to infer the molecular sex by comparing the number of alignments to the Y chromosome to the total number of alignments to X- and Y-chromosomes.

### 2.5. Y-Chromosome Haplogroup Determination

Y-chromosome haplogroup inference was attempted for male Neolithic samples using the software Yleaf v2.2 [24], which accepted read alignments in BAM format as input files**.** The prediction, performed with a minimum number of reads for each base (-r) set to 2 and a minimum quality for each read (-q) set to 30, was based on over 41,000 markers present in the International Society of Genetic Genealogy (ISOGG) Y-DNA Haplogroup Tree defining 5353 haplogroups [24].

### 2.6. Kinship Prediction

We first genotyped our read alignments in BAM format, after trimming 3 bases at read ends, by using bamUtil “trimBam” function (v1.0.14) [25], and SAMtools [26] “mpileup” function (v1.7), followed by pileupCaller from sequenceTools (v1.4.0.5, https://github.com/stschiff/sequenceTools, accessed on 1 August 2020) by randomly calling one allele per position considering the human genome as pseudo-haploid genome. Base alignment quality computation was disabled (-B) and minimum base (-q) and mapping quality (-Q) were set to 30 in the mpileup process, as recommended by the authors. We used GRCh37 as reference genome assembly and called the SNPs according to the 1240K panel [27,28,29]. Genotypes were obtained in Eigenstrat output format, subsequently converted in plink format using EIGENSOFT convertf (v7.2.1, https://github.com/DReichLab/EIG, accessed on 1 August 2020) and plink [30] (v1.9). Finally, kinship prediction was performed by READ [31], whose approach can successfully infer up to second degree relationships even with 0.1× shotgun coverage per genome for pairs of individuals. The analysis was carried out with default parameters, using the median across all pairwise differences for normalization.

In addition to READ, we also applied NgsRelate [32] (v2) to infer relatedness using genotype likelihoods instead of called genotypes. In this fashion, we compared kinship predictions obtained by two different approaches, useful for uncertain classifications due to low coverage. ANGSD [33] (v0.933) was used to calculate genotype likelihoods while doing SNP calling from the mapped reads with the GATK model [34], after trimming 3 bases from both read ends and requiring minimum base and mapping quality scores = 30. NgsRelate was run using European population allele frequencies, previously collected from the 1000 Genomes Project, phase 3 [35]. The tool calculated 11 summary statistics, including the coefficient of kinship theta [36], the KING-robust kinship statistics by Manichaikul et al. [37], and the “R0” and “R1” ratios described in Waples et al. [38], based on a two-dimensional site-frequency spectrum (2D-SFS) approach.

### 2.7. Analysis of Phenotypically Informative Markers

Variant calling of 41 SNPs from the HIrisPlex-S panel [39] related to eye, hair, and skin pigmentation was performed using two different approaches, allowing to get both pseudo-haploid and diploid calls on the same targeted sites. To the first aim, SAMtools mpileup and pileupCaller were used as described in the Section 2.6, while diploid calling was enabled by ATLAS [40] maximum likelihood genotype caller (task = call method = MLE) by specifying post-mortem damage and recalibration input files. These latter were previously obtained by ATLAS (“PMD” and “recal” functions, respectively) considering only bases with a minimum quality = 20 and using highly conserved positions among species defined by high GERP scores [41] as a training set to infer the recalibration parameters applied to our data. ATLAS allowed retrieving potential heterozygous variations, which could not be detected by the pseudo-haploid calling. We used the HIrisPlex-S [39,42,43,44] online web tool to predict eye, hair, and skin colour from DNA genotypes called by ATLAS. Moreover, 7 additional SNPs in *EDAR*, *MCM6*, *ABO*, and *RHCE* genes were also detected, being phenotypically informative markers. Annotations for each SNP identified in our samples were retrieved from SNPedia [45].

## 3. Results and Discussion

### 3.1. Mitochondrial Haplotype and Haplogroup Analysis

Our molecular study of the human skeletal remains from Kierzkowo first focused on the analysis of mitochondrial DNA (mtDNA) genomes obtained through a capture enrichment approach associated with next generation sequencing [3]. This strategy allowed us to obtain 15 complete or almost complete mtDNA profiles out of 17 samples. Polymorphisms according to rCRS are annotated in Table S1 and showed in a phylogenetic tree including information about haplogroup attribution (Figure S1). The C > T misincorporation percentages at 5′ termini and average fragment length as well as estimates of modern human contamination calculated for the mtDNA molecules are indicated in Table 1.

As expected, the reanalysis of haplogroup attributions based on a newer version of PhyloTree and HaploGrep agrees with previously published data [3], except for some samples now attributed to specific sub-lineages generally with a higher resolution (H28a instead of H28, U5b2b1a1 instead of U5b2b1, U5b1d1a instead of U5b1d1, U3b3 instead of U3b, and H1 instead of H1b for sample 3.1). Haplogroup H was the most frequently predicted and represented more than half of the mtDNA variation in our dataset with eight samples carrying three different lineages (H, H1 and H28a). Haplogroup U characterises four samples, three belonging to U5 lineages and one to U3b. The remaining three samples belong to haplogroups J, K, and W. At the haplotype level, despite some missing positions, four samples (corresponding to sample IDs 7.1, 8.4, 6.1, and 5.1) share the same mutation motif, which is attributed to the H28a haplogroup. This haplotype sharing could be explained (i) by a possible maternal relationship, more or less close in time, or (ii) by the presence of two or more samples belonging to the same individual (as shown below, further analyses at the nuclear genome level were necessary to support one of the two possible hypotheses).

The evaluation of damage patterns described through average fragment length and the C > T misincorporation percentage at 5′ termini showed a certain degree of molecular degradation for all the samples. Even if fragment length cannot be used as an antiquity marker (degradation of the DNA to an average size lower than 100 bp occurs rapidly after the death of the organism, probably due to autolytic processes [46,47]), the average length of our samples was estimated to range from 50.6 to 67.7 bp, in line with degradation processes.

Moreover, a different level of cytosine deamination at read ends was observed for the samples. Most of them showed percentages of C > T at 5′ ranging from 23% to 39% (values compatible with the Neolithic chronology of the site [46]), while three samples (6.2, 8.8, and 8.9) were characterised by lower percentages of molecules affected by misincorporation ranging from 11% to 12%.

As is known, opposite to fragment length, the frequency of misincorporations at the ends of the molecules tends to increase over time and can be used to distinguish modern from ancient DNA (aDNA) [46]. Therefore, the presence of a lower percentage of misincorporation for samples 6.2, 8.8, and 8.9 could be a signal of possible modern contamination of the samples or indicate that the three individuals did not belong to the Neolithic period.

The estimates obtained by the evaluation of ContamMix [22] allowed us to exclude a relevant contamination by modern human DNA for these samples. Referring to archaeological data, these three samples turned out to belong to a skeletal assemblage located outside the principal burial chamber of the tomb.

For these reasons, as described below, further investigation was required to explain the different damage patterns that characterise these samples.

### 3.2. Analysis of Nuclear Genome Data

Nuclear genome data were obtained through a target enrichment approach based on using 1240 K informative SNPs as a target [2]. The population genetics analysis showed the three individuals characterised by lower misincorporation percentages as diverging from the overall genetic variability associated with the other samples from Kierzkowo, providing further clues about a possible different provenance of these samples [2]. Radiocarbon dating was, therefore, applied to some of the bones (both with lower and higher values of misincorporation percentages) in order to directly check their chronology. Dating results confirmed the bones from the tomb chamber as belonging to the Globular Amphorae culture (GAC) period: 2870–2575 cal BCE (β-430,712, sample 3.4), 3260–3095 cal BCE (β-430,713, sample 8.2), and 3015 cal BCE (β-430,714, sample 8.4) [2]. Results obtained for the bones found outside the burial chamber proved, instead, that 6.2, 8.8, and 8.9 samples represented a recent intrusive burial (8.8 and 8.9 dated to 210 ± 30 BP and 130 ± 30 BP, respectively, β-430,716 and β-430,715 [2]) of an adult woman and two children (one infant and a 2–3 year-old child), who were not maternally related, according to the mtDNA profiles. As a result, archaeologists suggested that they may have been people who—for some reason—could not be buried in the “consecrated ground” or that they were victims of one of the epidemics that ravaged these lands, such as the plague in the early 18th century or the cholera epidemic in the 19th century. In fact, at some distance from Kierzkowo, a mass grave of the victims of the pestilence from the mid-19th century was discovered. In addition, several victims of the cholera epidemic were also buried in the 19th century in the megalithic tomb of the GAC in Złotowo, 10 km from Kierzkowo [11].

If data obtained from the study of mtDNA genomes first provided important information about possible relations among samples and their chronology, only with the analysis of nuclear DNA (nuDNA) data was it possible to clarify the clues arising from the mtDNA analysis and to obtain thorough attributions at the individual level. Nuclear genome analysis, indeed, allowed us to clarify the relationships among the four samples sharing the same mitochondrial H28a haplotype. By evaluating the mitochondrial and nuclear DNA data, it was possible to provide a proper estimate of the minimum number of individuals (MNI), which otherwise would not have been allowed due to the complex taphonomic situation of bone remains in the burial.

In addition, the comparison of the obtained nuDNA genotypes allowed us to identify and verify that samples 7.1 and 8.4 belonged to the same individual and aid the anthropological study in the correct site reconstruction since the two samples (a maxillary and femur fragment) had been previously tentatively attributed to different individuals because of surface appearance and state of preservation.

#### 3.2.1. Sex Identification

Nuclear genome data were obtained for eight individuals, of which six were identified as male and two as female (Table 2). The comparison of genetic and anthropological data highlighted a perfect match of results for all samples analysed, except sample 6.1 for which the genetic determination gave a result different from the anthropological estimation. Furthermore, it generally allowed us to obtain a determination in case of absence of diagnostic morphological markers, i.e., for highly fragmented remains and infant and juvenile individuals. As a result, genetic sex determination allowed us to improve the attributions previously made through the anthropological approach. Even in this case, the fragmentation and preservation level of skeletal material, as well as the presence of infant and juvenile individuals, represented a strong limit for proper sex determination via the anthropological approach.

#### 3.2.2. Y-Chromosome Haplogroup Determination

Three out of six male individuals yielded enough valid markers to allow a haplogroup determination with the approach used by Y-leaf 2.2 [24]. All three samples (3.4, 7.6, 8.5) belonged to haplogroup I2, with different levels of definition at the sub-haplogroup level (Table 3). As expected, the more valid markers were available per sample, the deeper attribution at the sub-haplogroup level was possible. Due to the limited number of shared covered positions between samples and their low coverage, it was not possible to perform a detailed comparison between individuals at the haplotype level. For this reason, we have been able to highlight the presence of the same haplogroup in more individuals but not go deeper into the estimate of haplotype sharing and possible kin relationships based on this uniparental marker.

#### 3.2.3. Kinship Analysis

**Figure 3:**
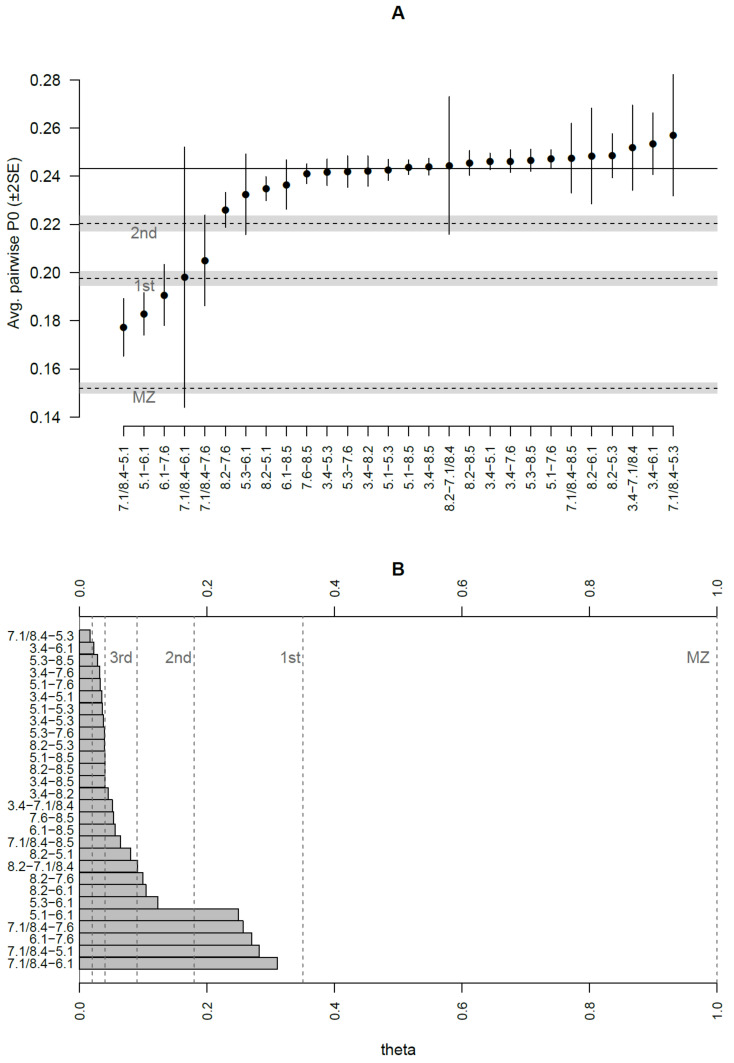
Kinship predictions by READ and NgsRelate software. (**A**) The average proportion of non-matching alleles (P0 values) with two standard errors calculated by READ is shown for each pair of samples. The solid horizontal line indicates the median value used for normalization. Dashed lines show the cut-offs calculated by READ to classify individuals as identical twins (MZ), first-degree, or second-degree related. The grey areas around dashed lines indicate 95% confidence intervals for the cut-offs. (**B**) Theta values for each pair of samples were calculated by NgsRelate. Dashed lines define ranges of expected theta values for each kinship category. We considered predictions of kinship by NgsRelate up to the third degree.

Kinship predictions by READ software [31] allowed us to identify the couples 6.1–7.6, 7.1/8.4–5.1, and 5.1–6.1 as possible parent–offspring or full-siblings, while 7.1/8.4–6.1 and 7.1/8.4–7.6 were classified as second-degree relatives (grandparent–grandchild, avuncular or half-siblings). However, the standard error of the average proportion of non-matching alleles (P0 values) calculated by READ [31] expressed very high uncertainty of the classification for the couple 7.1/8.4–6.1 (Figure 3), probably due to a reduced number of comparable genomic positions. To a lesser extent, the categorisation of most couples showed uncertainty, frequently spanning more than one class; thus, only the first-degree relationships involving sample 5.1 may be considered unambiguously predicted (Figure 3). We also performed a genotype likelihood-based analysis of kinship using NgsRelate [32] to compare kinship predictions obtained using different approaches (see Methods). Both methods led to quite similar outcomes and the full results are shown in Supplementary Table S2. First degree relationships involving 6.1 were confirmed and further distinguished using the R0 ratios described in Waples et al. [38] into parent–offspring or sibling relationships [38], resulting in a family unit composed of 5.1 as mother, 7.6 as father and 6.1 as son. A maternal relationship between mother and son was also confirmed by the same mtDNA profile belonging to haplogroup H28a. Theta values suggested a first degree of kinship for the couple 7.1/8.4–6.1; however, the prediction remained unreliable since only 189 genomic sites were considered in the analysis. A first-degree relationship was also estimated for the couple 7.1/8.4–7.6, differing from the READ prediction (second-degree). Low coverage, low degree of preservation of aDNA in these samples and possible inbreeding leading to a discrepancy from expected values of identity-by-descent (IBD), as shown for samples 7.1/8.4 and 6.1 (Table S2), could explain the difficulties in inferring kinship. Individual 7.1/8.4 could be, indeed, brother or uncle of 6.1, as well as brother of 5.1 as indicated also by mtDNA analysis; furthermore, he could be involved in a possible second-degree relationship with 7.6. Both biological and methodological reasons could complicate pedigrees, e.g., the previous generation showing some degree of relatedness or inaccurate population allele frequencies due to population structure in the data. The different kinship degree predicted by the two software programs could be explained by another possible reconstruction where individual 7.1/8.4 could be the half-brother of 6.1, (both sons of 5.1) with a possible kinship relation between their fathers.

Additional second-degree relationships could be inferred from the theta values involving 5.3 and 8.2 (although they were not confirmed by READ), as well as potential third-degree relationships for nine couples of samples. Moreover, the relationship for all the remaining pairs seemed to be more distant than that of third-degree, but it could be overestimated by NgsRelate, as shown for the old version of the tool [48]; thus, we considered these couples as unrelated. The KING statistic is frequently the most discordant among all the estimated predictors: it probably underestimates kinships, as already demonstrated [49].

**Figure 4:**
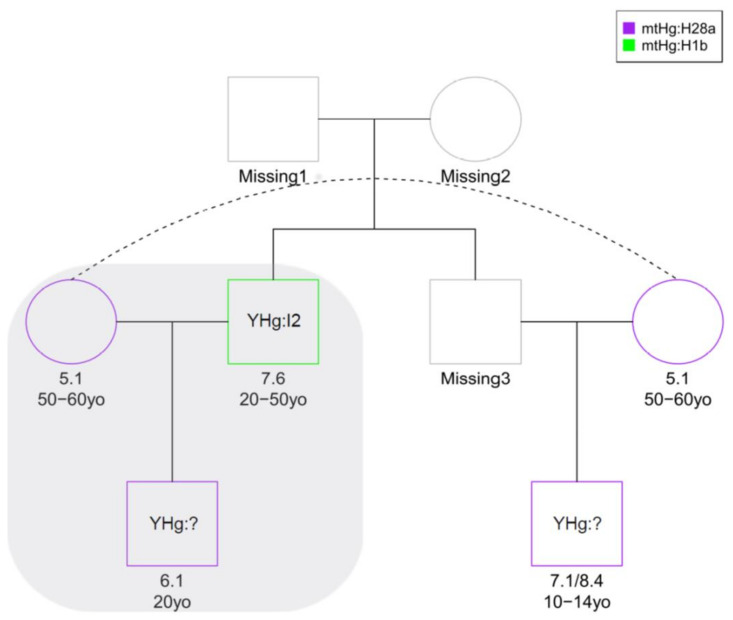
Pedigree reconstructed for the Kierzkowo family. A possible pedigree was inferred from genome-wide estimates of pairwise identity-by-descent (IBD). The nuclear family in the grey area was confirmed by both READ and NgsRelate tools. For each individual, age (yo: years old) estimated through morphological analysis is indicated. Y chromosome (YHg) haplogroup is described where available. Mitochondrial haplogroups (mtHg) are highlighted in different colours. Mitochondrial haplotype sharing supports the estimates of kinship relations between mother and sons based on genomic data (see Figure S1 and Table S1). The pedigree was plotted using the kinship2 R package [50].

As a result of kinship analysis, we were able to construct a reasonable possible pedigree tree including all four abovementioned samples by admitting the absence of some members of a hypothetical extended family among the remains (Figure 4), with the nuclear family composed of 5.1 (mother), 7.6 (father), and 6.1 (son), and considering 7.1/8.4–6.1 as half-siblings and 7.1/8.4–7.6 as nephew/uncle.

In an attempt to combine molecular with anthropological data, information about age classes estimated through morphological analysis could help to discriminate between different hypotheses about kinship relations, but we have to consider the uncertainty of the age estimates due to high fragmentation and degradation of skeletal remains and the possibility that individuals could have been dead and buried in different epochs.

Even if the obtained data did not allow a sound detailed reconstruction of the possible pedigrees, we can suppose the presence of at least a nuclear family and of different degrees of biological relationships between several couples of individuals buried in the Kierzkowo barrow. Even in this case, mtDNA data provided a first indication about possible relationships among individuals, but nuDNA information was necessary to discriminate between different possible reconstructions.

#### 3.2.4. Phenotypes

Because of low or no coverage, we were able to partially detect the selected phenotypically informative markers in our samples. Indeed, 32 of 41 HIrisPlex-S SNPs were not totally covered in some samples, presumably due to DNA fragmentation and because the remaining nine SNPs were not part of the 1240 K capture array. Generally, diploid genotyping (see Methods) called 35 out of the 48 analysed genomic positions (41 HIrisPlex-S + 7 additional markers), including three variants in a heterozygous state and 28 homozygous alleles (either reference or variant) confirmed by pseudo-haploid calling (Table S3). Samples 5.1, 6.1, and 8.5 probably had blue eyes, as predicted by the HIrisPlex-S tool [39], and confirmed by data reported in SNPedia 6 [45]. The same eye colour could be supposed for 3.4, 5.3, and 7.6 (SNPedia, Table S3). Hair colour was predicted for 5.1 (dark brown) and 8.5 (blonde), while 6.1 was probably light-haired. Red hair could be excluded as a phenotypic trait in more than half of the samples (3.4, 8.2, 5.3, in addition to 5.1 and 8.5). In terms of skin colour, pale skin was predicted for 8.5 and 5.1, which was also confirmed by SNPedia. The absence of freckles in 8.5 and light skin colour in 5.3 can be additionally supposed. Annotations by SNPedia are shown in Supplementary Table S3; probabilities calculated by the HIrisPlex-S tool are reported in Table 4 and Supplementary Table S4.

We also investigated *AB0* and *RHD* genes. Blood type 0 was inferred for 8.5, a classic OO homozygote like the 42% of Caucasians [45], while we could just exclude type B as the blood group for 3.4, 5.1, and 7.6, whose specific blood group could not be further determined due to a lack of coverage of the genomic position related to rs8176719 in the *ABO* gene. Similarly, Rhesus blood groups could not be properly inferred in our data since rs676785 and rs609320 in the *RHCE* gene (which determine c and e antigens, respectively) are not included in the 1240K SNPs panel. However, the *RHD* gene was partially covered in all the samples. Thus, its full deletion, which is mainly associated with the Rh-phenotype in Europeans [51], can be excluded.

Another trait we investigated is lactase persistence, since consumption of milk is attested in Kierzkowo by the presence of milk fats in ceramic vessels found in the burial and reconstruction of the diet through isotope analysis of human bone remains. Lactase is, indeed, the enzyme responsible for the digestion of the milk sugar lactose. Its production usually decreases after the weaning phase, except for some individuals who continue to produce lactase throughout adulthood. A mutation at the SNP rs4988235 (−13,910*T) in the *MCM6* gene, supporting the lactase persistence haplotype, explains the phenotype in European populations [52]. We were able to describe this SNP only in four individuals (5.1, 7.6, 8.2, and 8.5) from Kierzkowo. Interestingly, they all were homozygous for the ancestral allele, suggesting they could have become lactose intolerant in adulthood, probably with no effect on 8.5, who was a newborn. It is a common idea that lactase persistence coevolved with the cultural adaptation of dairying. Interestingly, the date estimates for the rise in frequency of the −13,910*T mutation (2188 and 20,650 years ago [53]) bracket archaeological dates for the spread of domestic animals and dairying into Europe, pointing at a probably strong positive selection linked to the cultural traits of animal domestication and adult milk consumption [53,54,55,56]. However, aDNA studies have shown that the −13,910*T allele was very rare or absent in early Neolithic central Europeans, which lets us suppose that lactase persistence and dairying coevolution began around 7500 years ago, probably among people of the Linearbandkeramik culture between the central Balkans and central Europe [57]. According to our data, people from Kierzkowo were lactose intolerant in adulthood, even though their culture was characterised by the importance of dairying. We have to consider that an inter-individual variation of the amount of lactose tolerated by lactase non-persistent people (probably as a result of variation in the composition of the gut flora) is known and that some intolerant individuals can consume lactose-containing products without any obvious ill effects. In particular, fermented dairy products (i.e., yoghurt or cheese) are usually well tolerated by non-persistent individuals since they contain less lactose [58].

### 3.3. Comparison between GAC Sites

A comparison of our results can be made with information available from another burial site attributed to the GAC in Poland, for which genome analysis was performed on human bone remains: the mass grave of Koszyce (Małopolska province) [59]. In this case, 15 individuals including men, women and children, were buried at the same time, next and on top of each other, after a violent death. According to the genomic data, the buried individuals belonged to a large extended family connected by several first- and second-degree relationships. In particular, four nuclear families were represented. The presence of unrelated females and related males would indicate a dominant form of patrilineal social organisation. The two burial contexts are quite different (a mass grave in Koszyce and a typical GAC burial ritual in Kierzkowo), as well as the experimental strategy applied due to a different level of sample preservation (whole genome sequencing for Koszyce samples and target enrichment of selected SNPs for Kierzkowo). However, similarly genetic and reproductive relationships were probably the basis of social relationships in GAC communities. In Kierzkowo as well as in Koszyce, the presence of different mtDNA lineages can support the hypothesis of a community not based on the matrilocal residence system. Unlike the Koszyce samples, the resolution of our data on the Y-chromosome did not allow us to confirm the presence of a unique patrilineal lineage, although we cannot exclude it either, since data for Kierzkowo show the same haplogroup for all the available samples. In particular, Y-chromosome variability is represented in both sites only by the I2 haplogroup, indicating a low genetic diversity in the GAC population according to this marker, which could support the hypothesis of a society based on patriarchy.

This hypothesis could find support from archaeological data: the economic base of the GAC group from Kierzkowo was agriculture and animal husbandry. In particular, the latter played a special role, as evidenced by the numerous skeletal remains of farm animals and the milk fat residues retrieved in ceramic vessels found in the burial, which suggest consumption of milk and meat. Preliminary isotope analysis on the human skeletal remains from Kierzkowo confirms the presence of these foods in the diet and seems to indicate better access to valuable animal products for men than for women [11]. Furthermore, most pastoralist societies are patriarchal due to the male role in animal husbandry. A particular role of men in GAC society is also sometimes demonstrated by the equipment of the graves, which is differentiated by gender [8,15,60,61,62,63].

## 4. Conclusions

Due to the (quite recent) introduction of next generation sequencing methodologies in the aDNA field, particular emphasis has been placed on archaeobiological research (archaeobotany, zooarchaeology, anthropology) as a way to gain insight into the environmental conditions and the social organisation of past populations and provide information at the individual level.

The study of the human bone remains found in the Kierzkowo Megalithic barrow allowed us to add important details to the description of this Neolithic community associated with the Globular Amphorae culture, and to the reconstruction of the use of the site through time. Indeed, the evaluation of nucleotide misincorporation patterns of aDNA (molecular degradation) suggested the presence—confirmed through radiocarbon C14 dating—of a recent intrusion into the burial that happened in the last century, when the remains of a woman and two little children were deposed inside the perimeter of the Neolithic burial. Together with the more recent remains, two female and six male Neolithic individuals were found in the tomb and identified. Despite the taphonomic condition of the site, characterised by a secondary deposition of fragmented and mixed bone remains, kin relationships and phenotypic traits of the buried individuals were also successfully estimated via aDNA analysis. Most of them were related by first-, second-, and third-degrees of kinship, and in particular, we located a nuclear family, one of the oldest attested by genetic data.

Considering the nuDNA data in general, the methodological approach used presented some limitations in terms of information recovered, as results are restricted to a relatively small and pre-selected set of genomic markers sequenced after a target enrichment. A whole-genome sequencing with high coverage would represent, indeed, the best strategy to obtain accurate individual profiling. However, producing whole-genome data is suitable for samples with levels of endogenous DNA content higher than that in Kierzkowo bone fragments (average 0.19%, with a minimum value of 0.007% and a maximum value of 0.66% for Neolithic individuals). More suitable samples than those used would have been represented by the petrous part of the temporal bone or by teeth, but they were not available for this study. Thus, to the best of our knowledge, the methodology chosen was the most suitable.

In conclusion, thanks to the development of the new sequencing technology and the choice of the best experimental strategy according to the level of preservation and the type of sample available, this study highlighted a new opportunity for ancient genomic studies.

Despite its limitations, even a target enrichment approach originally conceived for investigating large-scale populations’ structure and relationships can provide interesting information at the individual level that can be exploited for a fine reconstruction of singular archaeological contexts. Through a multidisciplinary approach that involves archaeologists, archaeobotanists, zooarchaeologists, and biological and molecular anthropologists, a detailed bioarchaeological reconstruction of ancient communities and their members can be supplied. From this perspective, molecular data represent an important informative source to support biological anthropological activity, answering specific questions and reconstructing individual profiles as well as biological and social relationships. The results presented in this study, together with possible further genetic data obtained for other GAC sites, can provide an important contribution that is useful for supporting archaeological and anthropological research on the social organisation and funerary rituals that characterised this culture.

## Figures and Tables

**Table 1:** The analysed specimens and summary of the results. Sample ID corresponds to the code attributed to the bone remains during the anthropological study, while Lab code corresponds to the laboratory label assigned during genotyping of nuclear genome. Note that Lab code is the same for sample 7.1 and 8.4, because the mitochondrial (mtDNA) and nuclear DNA (nuDNA) profiles indicated they belong to the same individual. Anatomical element, sex, and age determination obtained through anthropological methods [11,18] are indicated. Descriptive results for mtDNA and nuDNA analyses are reported. Direct radiocarbon dates are indicated where available. Samples 6.2, 6.3, 7.5, 8.8, and 8.9 were discarded from further analyses because of their low coverage or recent radiocarbon dating.

Sample ID	Lab Code nuDNA	Anatomical Element	Anthropological Sex and Age Estimation	mtDNA Molecules Average Length/ % CtoT at 5′ End	mtDNA Haplogroup	mtDNA Contamination Estimate (C.I.) %	SNPs Hit on Autosomes	Y-Chromosome Haplogroup	Molecular Sex Estimation	Conventional Radiocarbon Age	Calibrated Radiocarbon Result (95% Probability)
3.1	I2301	Left ulna	Male (50–X years)	67.6 bp/32	H1	6.56 (11.8–2.62)	-	-	-	-	-
3.4	I2403	Left femur	Male	63.8 bp/33	U5b2b1a1	0.08 (1.62–0.01)	154,174	I2a1b1	Male	4120 ± 30 BP (β-430,712)	Cal BC 2780 to 2575 (Cal BP 4730 to 4525)
5.1	I2433	Tooth/mandible	Female (50–60 years)	61.5 bp/39	H28a	3.89 (5.21–2.86)	528,330	-	Female	-	-
5.3b	I2434	Right femur	Female (20–30 years)	65.2 bp/32	U5b1d1a	1.31 (2.27–0.71)	112,399	-	Female	-	-
6.1	I2435	Femur	Female (20 years or more, closer to 20 years)	59.8 bp/30	H28a	0.00 (0.001–0.00)	16,888	NA	Male	-	-
6.2	-	Right ulna	Female over 25	67.0 bp/11	J1c2	0.39 (0.58–0.01)	406,352	-	-	-	Historical, based on the association with 8.8 and 8.9
6.3	-	Mandible	Female	61.2 bp/-	-	-	-	-	-	-	-
7.1	I2407	Maxilla	Juvenile (about 14 years)	61.0 bp/38	H28a	2.30 (2.62–2.04)	36,339	NA	Male	-	-
7.5	-	Right humerus	Juvenile (14–19 years)	54.3 bp/-	-	-	-	-	-	-	-
7.6	I2440	Right humerus	Male? (20–50 years)	66.3 bp/23	H1b	0.61 (1.13–0.29)	189,493	I2	Male	-	-
8.1	I2801	Right femur	Child (2–3 years)	56.9 bp/40	H1b	0.80 (0.89–0.67)	-	-	-	-	-
8.2	I2405	Tibia	Child (7–8 years)	50.9 bp/33	W5	2.30 (5.47–0.39)	91,505	NA	Male	4460 ± 30 BP (β-430,713)	Cal BC 3335 to 3210 (Cal BP 5285 to 5160), Cal BC 3190 to 3150 (Cal BP 5140 to 5100), Cal BC 3140 to 3020 (Cal BP 5090 to 4970)
8.3	I2803	Right tibia	Child (14 years)	66.7 bp/38	H	0.94 (2.67–0.20)	-	-	-	-	-
8.4	I2407	Left femur	Child (10–14 years)	64.2 bp/32	H28a	0.34 (1.63–0.03)	36,339	NA	Male	4390 ± 30 BP (β-430,713)	Cal BC 3095 to 2915 (Cal BP 5045 to 4865)
8.5	I2441	Left pelvis	Newborn	64.0 bp/31	K1b1a1	3.86 (4.19–3.50)	510,373	I2a1b1a2b1~	Male		
8.8	-	Left femur	Child (infant)	63.7 bp/11	U5b2a2b1	0.50 (1.05–0.07)	298,820	-	-	210 ± 30 BP (β-430,715)	Cal AD 1645 to 1685 (Cal BP 305 to 265), Cal AD 1735 to 1805 (Cal BP 215 to 145), Cal AD 1930 to Post 1950 (Cal BP 20 to Post 0)
8.9	-	Right femur	Child (2–3 years)	62.1 bp/12	U3b3	0.22 (2.71–0.02)	419,659	-	-	130 ± 30 BP (β-430,716)	Cal AD 1670 to 1780 (Cal BP 280 to 170), Cal AD 1800 to Post 1950 (Cal BP 150 to Post 0)

**Table 2:** Sex reconstruction for the Neolithic bones. Nseq: total number of sequences; NchrY + NchrX: total number of alignments to both sex chromosomes; NchrY: number of alignments to the Y chromosome; R_y: ratio between NchrY and NchrY + NchrX; SE: R_y standard error; 95% CI: 95% confidence interval for R_y.

Sample ID	Nseqs	NchrY + NchrX	NchrY	R_y	SE	95% CI	Assignment
3.4	690,382	18,622	5780	0.3104	0.0034	0.3037–0.317	XY
5.1	2,074,197	74,852	334	0.0045	0.0002	0.004–0.0049	XX
5.3	267,061	9152	59	0.0064	0.0008	0.0048–0.0081	XX
6.1	37,173	979	284	0.2901	0.0145	0.2617–0.3185	XY
7.1/8.4	19,211	522	155	0.2969	0.02	0.2577–0.3361	XY
7.6	382,899	10,099	3389	0.3356	0.0047	0.3264–0.3448	XY
8.2	195,536	5291	1663	0.3143	0.0064	0.3018–0.3268	XY
8.5	1,783,600	48,164	14,351	0.298	0.0021	0.2939–0.302	XY

**Table 3:** Y-chromosome haplogroup determination. Hg: final haplogroup predicted using ISOGG nomenclature. Hg_Marker: final haplogroup predicted using marker nomenclature. Total_Reads: total number of reads. Valid_Markers: number of markers used for the haplogroups prediction. QC_score: overall quality score that is the factor of the three scores QC-1, QC-2, and QC-3; QC-1: score that indicates whether the predicted haplogroup follows the expected backbone of the haplogroup tree structure; QC-2: score that indicates whether equivalent markers to the final haplogroup prediction were found in the ancestral state; QC-3: score that indicates whether the predicted haplogroup follows the expected within haplogroup tree structure.

Sample ID/Lab Code	Hg	Hg _Marker	Total_Reads	Valid_Markers	QC-Score	QC-1	QC-2	QC-3
3.4	I2a1b1	I-FGC3552/etc*(xY10631,Y33740,Y7225, A11214,Y5728,Y11154,Y16445,Y5224,Y6098)	527,527	526	1.0	1.0	1.0	1.0
6.1	NA	NA	41,862	11	0	0	0	0
7.1/8.4	NA	NA	21,520	7	0	0	0	0
7.6	I2	I-CTS8444*(xPF4049,Y22356,Y24165,Y7822,Y5728,S17264,Y68424,Z45444)	307,275	274	1.0	1.0	1.0	1.0
8.2	NA	NA	175,789	48	0	0	0	0
8.5	I2a1b1a2b1~	I-FGC3587*(xFGC3570)	1,056,530	2025	1.0	1.0	1.0	1.0

**Table 4:** HIrisPlex-S annotations and probabilities. Prediction probabilities calculated by the HIrisPlex-S software are shown only for the three samples with available data. Complete results are provided in Supplementary Table S4. In terms of eye colour, the predicted phenotype (blue) was defined by the highest p-values. In terms of hair colour, the highest p-value approach is used in combination with a stepwise model as described in [43]. In terms of skin colour, the highest p-value approach is used in combination with the second highest probability value [39].

Sample ID	Blue Eye	Intermediate Eye	Brown Eye	Blond Hair	Brown Hair	Red Hair	Black Hair	Light Hair	Dark Hair	Very Pale Skin	Pale Skin	Intermediate Skin	Dark Skin	Dark to Black Skin
5.1	0.898	0.063	0.039	0.371	0.467	0.008	0.154	0.779	0.221	0.081	0.539	0.380	0.000	0.000
6.1	0.912	0.057	0.031	NA	NA	NA	NA	0.934	0.066	NA	NA	NA	NA	NA
8.5	0.914	0.055	0.031	0.651	0.216	0.113	0.020	0.953	0.047	0.123	0.656	0.220	0.000	0.000

## Data Availability

The data analysed in this study are openly available in the European Nucleotide Archive (ENA) under accession numbers ERS2040835-ERS2040840, ERS2040844, and ERS2040845.

## Supplementary Materials

The following are available online at https://www.mdpi.com/article/10.3390/genes12060910/s1, Table S1: Polymorphisms according to rCRS and Mitochondrial Haplogroup determination, Table S2: Kinship analysis by READ and NgsRelate, Table S3: Annotation of phenotypically informative SNPs detected by ATLAS in the dataset, Table S4: Eye, hair, and skin colour prediction according to HIrisPlex-S on SNPs detected by ATLAS, Figure S1: Phylogenetic tree and haplogroup determination for fifteen mitogenomes from Kierzkowo. Polymorphisms are indicated according to rCRS reference sequence. Haplogroup assignment is based on PhyloTree Build 17. For the sake of simplicity, sample IDs were used to indicate samples.

## References

[1] Nielsen R., Akey J.M., Jakobsson M., Pritchard J.K., Tishkoff S., Willerslev R.N.E. (2017). Tracing the peopling of the world through genomics. Nature.

[2] Mathieson I., Alpaslan-Roodenberg S., Posth C., Szecsenyi-Nagy A., Rohland N., Mallick S., Olalde I., Broomandkhoshbacht N., Candilio F., Cheronet O. (2018). The genomic history of southeastern Europe. Nature.

[3] Tassi F., Vai S., Ghirotto S., Lari M., Modi A., Pilli E., Brunelli A., Susca R.R., Budnik A., Labuda D. (2017). Genome diversity in the Neolithic Globular Amphorae culture and the spread of Indo-European languages. Proc. R. Soc. B Biol. Sci..

[4] Veeramah K.R. (2018). The importance of fine-scale studies for integrating paleogenomics and archaeology. Curr. Opin. Genet. Dev..

[5] Johnson K.M., Paul K.S. (2016). Bioarchaeology and kinship: integrating theory, social relatedness, and biology in ancient family research. J. Archaeol. Res..

[6] Vai S., Amorim C.E.G., Lari M., Caramelli D. (2020). Kinship determination in archeological contexts through DNA analysis. Front. Ecol. Evol..

[7] Fortes G.G., Speller C.F., Hofreiter M., King T.E. (2013). Phenotypes from ancient DNA: Approaches, insights and prospects. Bioessays.

[8] Hensel W. (1980). Polska Starożytna.

[9] Wiślański T. (1966). Kultura Amfor Kulistych w Polsce Północno-Zachodniej.

[10] Wiślański T., Hensel W., Wiślański T. (1979). Dalszy rozwój ludów neolitycznych. Plemiona kultury amfor kulistych. Prahistoria Ziem Polskich.

[11] Nowaczyk S., Pospieszny Ł., Sobkowiak-Tabaka I. (2017). A Megalityczny grobowiec kultury amfor kulistych z Kierzkowa: Milczący świadek kultu przodków z epoki kamienia. Biskup. Archaeol. Work..

[12] Bakker J.A. (1992). The Ditch Hunebedden. Megalithic Tombs of the Funnel Beaker Culture.

[13] Wiślański T. (1982). Kierzkowo, gm. Jadowniki, woj. Bydgoskie. Stanowisko. Sprawozdanie z 1982 Roku.

[14] Wiślański T. (1982). Kierzkowo, stan. 1, gm. Jadowniki k. Żnina. Sprawozdanie z 1982 roku.

[15] Ciesielska A. (2011). Społeczeństwa Europy Pradziejowej.

[16] Gąssowski J. (1985). Kultura Pradziejowa na Ziemiach Polski.

[17] Godłowski K., Kozłowski J.K. (1976). Historia Starożytna Ziem Polskich.

[18] Budnik A., Wrzesiński J., Wrzesiński J. (2002). Kierzkowo—Między inhumacją a ciałopaleniem. Popiół i Kość.

[19] Weissensteiner H., Pacher D., Kloss-Brandstatter A., Forer L., Specht G., Bandelt H.J., Kronenberg F., Salas A., Schonherr S. (2016). HaploGrep 2: Mitochondrial haplogroup classification in the era of high-throughput sequencing. Nucleic Acids Res..

[20] Van Oven M., Kayser M. (2009). Updated comprehensive phylogenetic tree of global human mitochondrial DNA variation. Hum. Mutat..

[21] Jónsson H., Ginolhac A., Schubert M., Johnson P.L.F., Orlando L. (2013). mapDamage2.0: Fast approximate Bayesian estimates of ancient DNA damage parameters. Bioinformatics.

[22] Fu Q., Mittnik A., Johnson P.L.F., Bos K., Lari M., Bollongino R., Sun C., Giemsch L., Schmitz R., Burger J. (2013). A revised timescale for human evolution based on ancient mitochondrial genomes. Curr. Biol..

[23] Skoglund P., Storå J., Götherström A., Jakobsson M. (2013). Accurate sex identification of ancient human remains using DNA shotgun sequencing. J. Archaeol. Sci..

[24] Ralf A., Gonzalez D.M., Zhong K., Kayser M. (2018). Yleaf: Software for human Y-chromosomal haplogroup inference from next-generation sequencing data. Mol. Biol. Evol..

[25] Jun G., Wing M.K., Abecasis G.R., Kang H.M. (2015). An efficient and scalable analysis framework for variant extraction and refinement from population-scale DNA sequence data. Genome Res..

[26] Li H., Handsaker B., Wysoker A., Fennell T., Ruan J., Homer N. (2009). The sequence alignment/map format and SAMtools. Bioinformatics.

[27] Fu Q., Hajdinjak M., Moldovan O.T., Constantin S., Mallick S., Skoglund P., Patterson N., Rohland N., Lazaridis I., Nickel B. (2015). An early modern human from Romania with a recent Neanderthal ancestor. Nature.

[28] Haak W., Lazaridis I., Patterson N., Rohland N., Mallick S., Llamas B., Brandt G., Nordenfelt S., Harney E., Stewardson K. (2015). Massive migration from the steppe was a source for Indo-European languages in Europe. Nature.

[29] Lazaridis I., Nadel D., Rollefson G., Merrett D.C., Rohland N., Mallick S., Fernandes D., Novak M., Gamarra B., Sirak K. (2016). Genomic insights into the origin of farming in the ancient Near East. Nature.

[30] Purcell S., Neale B., Todd-Brown K., Thomas L., Ferreira M.A.R., Bender D., Maller J., Sklar P., de Bakker P.I.W., Daly M.J. (2007). PLINK: A tool set for whole-genome association and population-based linkage analyses. Am. J. Hum. Genet..

[31] Kuhn J.M.M., Jakobsson M., Günther T. (2018). Estimating genetic kin relationships in prehistoric populations. PLoS ONE.

[32] Hanghøj K., Moltke I., Andersen P.A., Manica A., Korneliussen T.S. (2019). Fast and Accurate Relatedness Estimation from High-throughput Sequencing Data in the Presence of Inbreeding. Gigascience.

[33] Korneliussen T.S., Albrechtsen A., Nielsen R. (2014). ANGSD: Analysis of next generation sequencing data. BMC Bioinform..

[34] McKenna A., Hanna M., Banks E., Sivachenko A., Cibulskis K., Kernytsky A. (2010). The genome analysis toolkit: A MapReduce framework for analyzing next-generation DNA sequencing data. Genome Res..

[35] Genomes Project C., Auton A., Brooks L.D., Durbin R.M., Garrison E.P., Kang H.M., Korbel J.O., Marchini J.L., McCarthy S., McVean G.A. (2015). A global reference for human genetic variation. Nature.

[36] Jacquard A. (1974). The Genetic Structure of Populations.

[37] Manichaikul A., Mychaleckyj J.C., Rich S.S., Daly K., Sale M., Chen W.-M. (2010). Robust relationship inference in genome-wide association studies. Bioinformatics.

[38] Waples R.K., Albrechtsen A., Moltke I. (2019). Allele frequency-free inference of close familial relationships from genotypes or low-depth sequencing data. Mol. Ecol..

[39] Chaitanya L., Breslin K., Zuñiga S., Wirken L., Pośpiech E., Kukla-Bartoszek M., Sijen T., de Knijff P., Liu F., Branicki W. (2018). The HIrisPlex-S system for eye, hair and skin colour prediction from DNA: Introduction and forensic developmental validation. Forensic Sci. Int. Genet..

[40] Link V., Kousathanas A., Veeramah K., Sell C., Scheu A., Wegmann D. (2017). ATLAS: Analysis tools for low-depth and ancient samples. BioRxiv.

[41] Davydov E.V., Goode D.L., Sirota M., Cooper G.M., Sidow A., Batzoglou S. (2010). Identifying a high fraction of the human genome to be under selective constraint using GERP++. PLoS Comput. Biol..

[42] Walsh S., Chaitanya L., Breslin K., Muralidharan C., Bronikowska A., Pospiech E., Koller J., Kovatsi L., Wollstein A., Branicki W. (2017). Global skin colour prediction from DNA. Hum. Genet..

[43] Walsh S., Chaitanya L., Clarisse L., Wirken L., Draus-Barini J., Kovatsi L., Maeda H., Ishikawa T., Sijen T., de Knijff P. (2014). Developmental validation of the HIrisPlex system: DNA-based eye and hair colour prediction for forensic and anthropological usage. Forensic Sci. Int. Genet..

[44] Walsh S., Wollstein A., Liu F., Chakravarthy U., Rahu M., Seland J.H., Soubrane G., Tomazzoli L., Topouzis F., Vingerling J.R. (2012). DNA-based eye colour prediction across Europe with the IrisPlex system. Forensic Sci. Int. Genet..

[45] Cariaso M., Lennon G. (2012). SNPedia: A wiki supporting personal genome annotation, interpretation and analysis. Nucleic Acids Res..

[46] Sawyer S., Krause J., Guschanski K., Savolainen V., Pääbo S. (2012). Temporal patterns of nucleotide misincorporations and DNA fragmentation in ancient DNA. PLoS ONE.

[47] Paabo S. (1989). Ancient DNA: Extraction, characterization, molecular cloning, and enzymatic amplification. Proc. Natl. Acad. Sci. USA.

[48] Sikora M., Seguin-Orlando A., Sousa V.C., Albrechtsen A., Korneliussen T., Ko A., Rasmussen S., Dupanloup I., Nigst P.R., Bosch M.D. (2017). Ancient genomes show social and reproductive behavior of early Upper Paleolithic foragers. Science.

[49] Dou J., Sun B., Sim X., Hughes J.D., Reilly D.F., Tai E.S., Liu J., Wang C. (2017). Estimation of kinship coefficient in structured and admixed populations using sparse sequencing data. PLoS Genet..

[50] Sinnwell J.P., Therneau T.M., Schaid D.J. (2014). The kinship2 R package for pedigree data. Hum. Hered..

[51] Avent N.D., Reid M.E. (2000). The Rh blood group system: A review. Blood.

[52] Itan Y., Jones B.L., Ingram C.J., Swallow D.M., Thomas M.G. (2010). A worldwide correlation of lactase persistence phenotype and genotypes. BMC Evol. Biol..

[53] Bersaglieri T., Sabeti P.C., Patterson N., Vanderploeg T., Schaffner S.F., Drake J.A., Rhodes M., Reich D.E., Hirschhorn J.N. (2004). Genetic signatures of strong recent positive selection at the lactase gene. Am. J. Hum. Genet..

[54] Gerbault P., Liebert A., Itan Y., Powell A., Currat M., Burger J., Swallow D.M., Thomas M.G. (2011). Evolution of lactase persistence: An example of human niche construction. Philos. Trans. R. Soc. Lond. Ser. B Biol. Sci..

[55] Evershed R.P., Payne S., Sherratt A.G., Copley M.S., Coolidge J., Urem-Kotsu D., Kotsakis K., Özdoğan M., Özdoğan A.E., Nieuwenhuyse O. (2008). Earliest date for milk use in the Near East and southeastern Europe linked to cattle herding. Nature.

[56] Pinhasi R., Fort J., Ammerman A.J. (2005). Tracing the origin and spread of agriculture in Europe. PLoS Biol..

[57] Itan Y., Powell A., Beaumont M.A., Burger J., Thomas M.G. (2009). The Origins of lactase persistence in Europe. PLoS Comput. Biol..

[58] Lomer M.C., Parkes G.C., Sanderson J.D. (2008). Review article: Lactose intolerance in clinical practice—Myths and realities. Aliment. Pharm..

[59] Schroeder H., Margaryan A., Szmyt M., Theulot B., Wlodarczak P., Rasmussen S., Gopalakrishnan S., Szczepanek A., Konopka T., Jensen T.Z.T. (2019). Unraveling ancestry, kinship, and violence in a Late Neolithic mass grave. Proc. Natl. Acad. Sci. USA.

[60] Sałacińska B., Sałaciński S. (2010). Rewolucja neolityczna na Mazowszu: Początki nowoczesnej gospodarki, Mazowsze. Studia Reg..

[61] Szmyt M. (2002). Kugelamphoren-Gemeinschaften in Mittel- und Osteuropa: Siedlungs-Strukturen und Soziale Fragen. UPA.

[62] Szmyt M., Kośko A., Szmyt M. (2004). Wędrówki bliskie i dalekie. Ze studiów nad organizacją społeczną i gospodarką ludności kultury amfor kulistych na terenie Europy Środkowej i Wschodniej. Nomadyzm i Pastoralizm w Międzyrzeczu Wisły i Dniepru (Neolit, Eneolit, Epoka Brązu), Archeologia Bimaris. Dyskusje 3.

[63] Woidich M. (2014). Die Westliche Kugelamphorenkultur: Untersuchungen zu Ihrer Raumzeitlichen Differenzierung, Kulturellen und Anthropologischen Identitét.

