# Supplementary figures and images for: How a Paleogenomic Approach Can Provide Details on Bioarchaeological Reconstruction: A Case Study from the Globular Amphorae Culture
Source: Genes (Basel). 2021 Jun 11;12(6):910. doi: 10.3390/genes12060910 (PMC8230892; doi:10.3390/genes12060910)

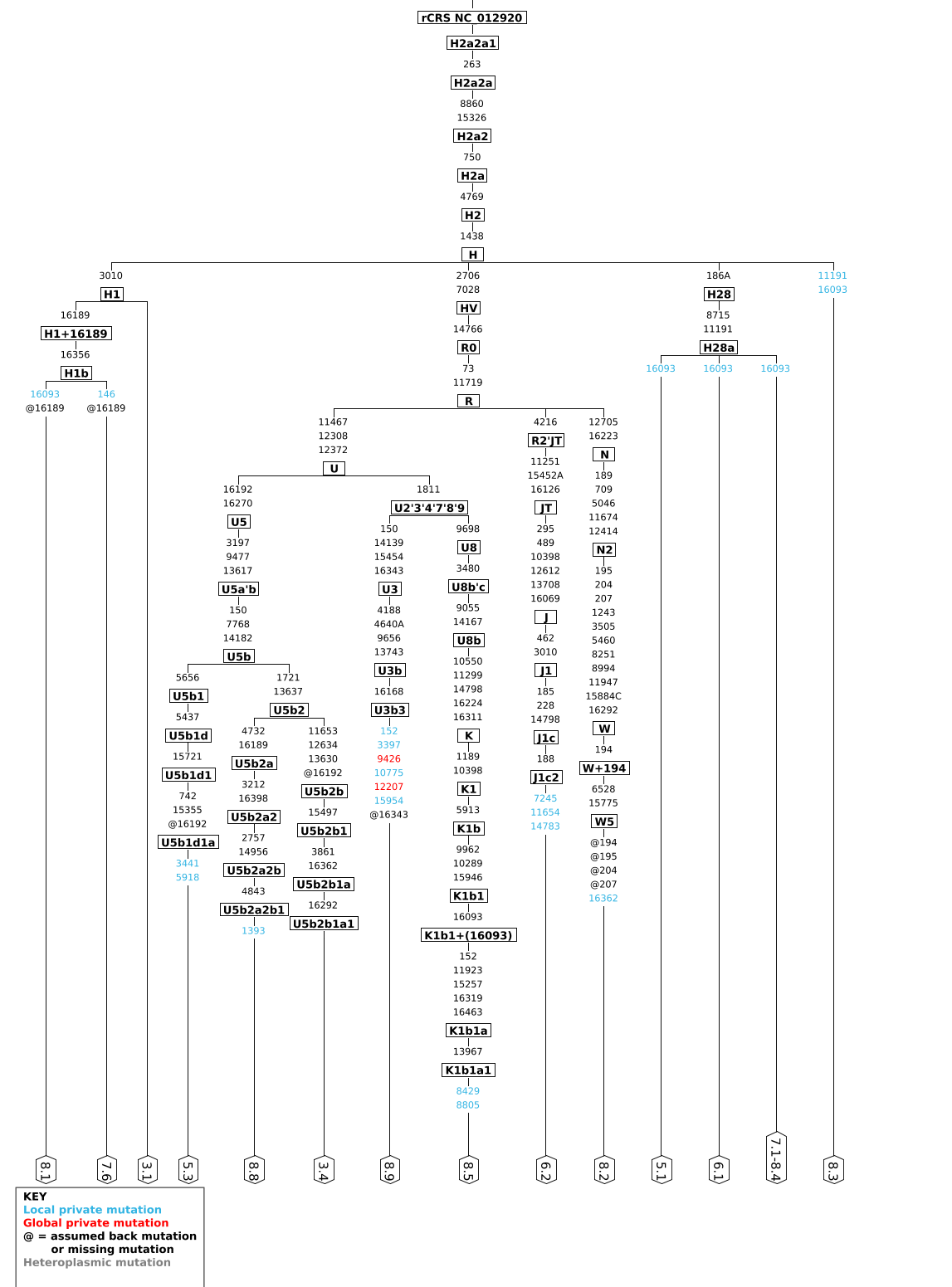

Supplement: Supplementary file 1 [file genes-12-00910-s001.zip › Figure_S1.png]
